# Supplementary material for: Effects of grazing strategy on facultative grassland bird nesting on native grassland pastures of the Mid-South USA
Source: PeerJ. 2022 Sep 28;10:e13968. doi: 10.7717/peerj.13968 (PMC9526412; doi:10.7717/peerj.13968)
Supplement: Table S1 [file peerj-10-13968-s003.docx]

Supplemental Table 1

Grassland bird nests found on rotational grazed (ROT), patch-burn grazed (PBG), and pre-treatment (Pre) pastures at 3 different research sites (BGAD, DREC, and QUICK) to assess the impacts of each method on nest survival and nest-site selection in the Mid-South USA from 2014 – 2016.

|  | BGAD | | | DREC | | | QUICK | | | Total | | | Grand Total |
| --- | --- | --- | --- | --- | --- | --- | --- | --- | --- | --- | --- | --- | --- |
| Species | Pre | PBG | ROT | Pre | PBG | ROT | Pre | PBG | ROT | Pre | PBG | ROT |  |
| Wild turkey | 0 | 0 | 0 | 0 | 2 | 0 | 0 | 0 | 1 | 0 | 2 | 1 | 3 |
| (*Meleagris gallapavo*) |  |  |  |  |  |  |  |  |  |  |  |  |  |
| Eastern kingbird | 0 | 0 | 0 | 1 | 2 | 0 | 0 | 0 | 0 | 1 | 2 | 0 | 3 |
| (*Tyrannus tyrannus*) |  |  |  |  |  |  |  |  |  |  |  |  |  |
| Carolina wren | 0 | 0 | 0 | 0 | 1 | 0 | 0 | 0 | 0 | 0 | 1 | 0 | 1 |
| (*Thryothorus ludovicianus*) |  |  |  |  |  |  |  |  |  |  |  |  |  |
| Brown thrasher | 0 | 0 | 0 | 0 | 0 | 1 | 0 | 0 | 0 | 0 | 1 | 0 | 1 |
| (*Toxostoma rufum*) |  |  |  |  |  |  |  |  |  |  |  |  |  |
| Eastern bluebird | 0 | 0 | 0 | 0 | 1 | 0 | 0 | 0 | 0 | 0 | 0 | 0 | 1 |
| (*Sialia sialis*) |  |  |  |  |  |  |  |  |  |  |  |  |  |
| American goldfinch | 0 | 0 | 0 | 1 | 0 | 0 | 0 | 0 | 0 | 1 | 0 | 0 | 1 |
| (*Spinus tristis*) |  |  |  |  |  |  |  |  |  |  |  |  |  |
| Field sparrow* | 14 | 8 | 12 | 26 | 68 | 38 | 1 | 8 | 6 | 41 | 84 | 56 | 181 |
| (*Spizella pusilla*) |  |  |  |  |  |  |  |  |  |  |  |  |  |
| Grasshopper sparrow | 0 | 0 | 0 | 6 | 0 | 0 | 5 | 7 | 5 | 11 | 7 | 5 | 23 |
| (*Ammodramus savannarum*) |  |  |  |  |  |  |  |  |  |  |  |  |  |
| Henslow's sparrow | 5 | 0 | 1 | 0 | 0 | 0 | 0 | 0 | 0 | 5 | 0 | 1 | 6 |
| (*Ammodramus henslowii*) |  |  |  |  |  |  |  |  |  |  |  |  |  |
| Song sparrow | 0 | 0 | 0 | 0 | 1 | 1 | 0 | 0 | 0 | 0 | 1 | 1 | 2 |
| (*Melospiza melodia*) |  |  |  |  |  |  |  |  |  |  |  |  |  |
| Chipping sparrow | 0 | 0 | 0 | 2 | 0 | 0 | 0 | 0 | 0 | 2 | 0 | 0 | 2 |
| (*Spizella passerine*) |  |  |  |  |  |  |  |  |  |  |  |  |  |
| Yellow-breasted chat | 0 | 0 | 0 | 0 | 0 | 0 | 0 | 1 | 0 | 0 | 1 | 0 | 1 |
| (*Icteria virens*) |  |  |  |  |  |  |  |  |  |  |  |  |  |
| Red-winged blackbird* | 8 | 1 | 8 | 10 | 0 | 0 | 0 | 1 | 6 | 18 | 2 | 14 | 34 |
| (*Agelaius phoeniceus*) |  |  |  |  |  |  |  |  |  |  |  |  |  |
| Eastern meadowlark | 0 | 0 | 0 | 4 | 0 | 1 | 0 | 0 | 0 | 4 | 0 | 1 | 5 |
| (*Strunella magna*) |  |  |  |  |  |  |  |  |  |  |  |  |  |
| Common yellowthroat | 1 | 5 | 6 | 0 | 0 | 1 | 0 | 0 | 0 | 1 | 5 | 7 | 13 |
| (*Geothlypis trichas*) |  |  |  |  |  |  |  |  |  |  |  |  |  |
| Prairie warbler | 0 | 0 | 0 | 0 | 0 | 0 | 0 | 0 | 1 | 0 | 0 | 1 | 1 |
| (*Setophaga discolor*) |  |  |  |  |  |  |  |  |  |  |  |  |  |
| Indigo bunting* | 0 | 13 | 6 | 13 | 3 | 1 | 0 | 7 | 1 | 13 | 23 | 8 | 44 |
| (*Passerina cyanea*) |  |  |  |  |  |  |  |  |  |  |  |  |  |
| Northern cardinal | 0 | 1 | 0 | 1 | 1 | 1 | 0 | 0 | 0 | 1 | 2 | 1 | 4 |
| (*Cardinalis cardinalis*) |  |  |  |  |  |  |  |  |  |  |  |  |  |
| Blue grosbeak | 0 | 0 | 1 | 5 | 1 | 0 | 0 | 0 | 0 | 5 | 1 | 1 | 7 |
| (*Passerina caerulea*) |  |  |  |  |  |  |  |  |  |  |  |  |  |
| Dickcissel | 0 | 0 | 0 | 1 | 0 | 0 | 0 | 0 | 0 | 1 | 0 | 0 | 1 |
| (*Spiza americana*) |  |  |  |  |  |  |  |  |  |  |  |  |  |
| Total | 28 | 28 | 34 | 70 | 80 | 44 | 6 | 24 | 20 | 104 | 132 | 98 | 334 |

*indicates selected grassland bird for analysis during the study.
